# Reliability of cardiac output measurements using LiDCOrapid™ and FloTrac/Vigileo™ across broad ranges of cardiac output values

**DOI:** 10.1007/s10877-016-9896-7

**Published:** 2016-06-14

**Authors:** Masaaki Asamoto, Ryo Orii, Mikiya Otsuji, Masahiko Bougaki, Yousuke Imai, Yoshitsugu Yamada

**Affiliations:** 1 Department of Anesthesiology and Pain Relief Center, The University of Tokyo Hospital, Tokyo, Japan; 2 Department of Anesthesiology, Tokyo Teishin Hospital, Tokyo, Japan

**Keywords:** Cardiac output, Systemic vascular resistance, Monitoring, Low invasive instruments, Flotrac, LiDCOrapid, Pulse wave analysis, Thermodilution, Pulmonary artery catheter

## Abstract

Knowing a patient’s cardiac output (CO) could contribute to a safe, optimized hemodynamic control during surgery. Precise CO measurements can serve as a guide for resuscitation therapy, catecholamine use, differential diagnosis, and intervention during a hemodynamic crisis. Despite its invasiveness and intermittent nature, the thermodilution technique via a pulmonary artery catheter (PAC) remains the clinical gold standard for CO measurements. LiDCOrapid™ (LiDCO, London, UK) and FloTrac/Vigileo™ (Edwards Lifesciences, Irvine, CA) are less invasive continuous CO monitors that use arterial waveform analysis. Their calculations are based on arterial waveform characteristics and do not require calibration. Here, we evaluated LiDCOrapid™ and FloTrac/Vigileo™ during off-pump coronary artery bypass graft (OPCAB) and living-donor liver transplantation (LDLT) surgery. This observational, single-center study included 21 patients (11 OPCAB and 10 LDLT). We performed simultaneous measurements of CO at fixed sampling points during surgery using both devices (LiDCOrapid™ version 1.04-b222 and FloTrac/Vigileo™ version 3.02). The thermodilution technique via a PAC was used to obtain the benchmark data. LiDCOrapid™ and FloTrac/Vigileo™ were used in an uncalibrated fashion. We analyzed the measured cardiac index using a Bland–Altman analysis (the method of variance estimates recovery), a polar plot method (half-moon method), a 4-quadrant plot and compared the widths of the limits of agreement (LOA) using an *F* test. One OPCAB patient was excluded because of the use of an intra-aortic balloon pumping during surgery, and 20 patients (10 OPCAB and 10 LDLT) were ultimately analyzed. We obtained 149 triplet measurements with a wide range of cardiac index. For the FloTrac/Vigileo™, the bias and percentage error were −0.44 L/min/m^2^ and 74.4 %. For the LiDCOrapid™, the bias and percentage error were −0.38 L/min/m^2^ and 53.5 %. The polar plot method showed an angular bias (FloTrac/Vigileo™ vs. LiDCOrapid™: 6.6° vs. 5.8°, respectively) and radial limits of agreement (−63.9 to 77.1 vs. −41.6 to 53.1). A 4-quadrant plot was used to obtain concordance rates (FloTrac/Vigileo™ vs. PAC and LiDCOrapid™ vs. PAC: 84.0 and 92.4 %, respectively). We could compare CO measurement devices across broad ranges of CO and SVR using LDLT and OPCAB surgical patients. An *F* test revealed no significant difference in the widths of the LoA for both devices when sample sizes capable of detecting a more than two-fold difference were used. We found that both devices tended to underestimate the calculated CIs when the CIs were relatively high. These proportional bias produced large percentage errors in the present study.

## Introduction

Knowing a patient’s cardiac output (CO) could contribution to safe, optimized hemodynamic control during surgery. Precise CO measurements can serve as a guide for resuscitation therapy, catecholamine use, differential diagnosis, and intervention during a hemodynamic crisis [1, 2]. Although the thermodilution technique via a pulmonary artery catheter (PAC) has an invasive and intermittent nature, it still remains the clinical gold standard for CO measurements [3]. LiDCOrapid™ (LiDCO, London, UK) and FloTrac/Vigileo™ (Edwards Lifesciences, Irvine, CA) are examples of less invasive and continuous CO monitors that use an arterial waveform analysis. Their calculations are based on arterial waveform characteristics and do not require calibration. The evaluation and comparison of these low-invasion CO estimation monitors are invaluable for the rational selection of CO measurement methods [3]. Many research projects have been performed in this field [4–8], but most studies were implemented across narrow ranges of cardiac output and systemic vascular resistance values, and did not compare the accuracy of the new devices directly.

This present study focused on the use of live-donor liver transplant (LDLT) recipients and off-pump coronary artery bypass graft (OPCAB) cases for comparing the devices. LDLT recipients exhibit a hyperdynamic state in the presences of cirrhosis, with a low systemic vascular resistance (SVR) and a high CO [9, 10]. The OPCAB cases, on the other hand, have a high SVR due to arteriosclerosis and a low CO due to myocardial ischemia [11, 12]. We assumed that, using LDLT and OPCAB surgical patients, we would be able to compare CO measurement devices across broad ranges of CO and SVR.

## Methods

This research was approved by the ethics committee of the University of Tokyo Hospital (#3926). All the patients provided written informed consent.

### Sample size determination

The number of enrolled patients was determined using a power analysis so as to detect a more than two-fold differences in the width of the limits of agreement (LoA). Using the reported terms described by Zou [13], the conditions were described as shown below:

$${\raise0.7ex\hbox{${s_{tot ft}^{2} }$} \!\mathord{\left/ {\vphantom {{s_{tot ft}^{2} } {s_{tot lid}^{2} }}}\right.\kern-0pt} \!\lower0.7ex\hbox{${s_{tot lid}^{2} }$}} < \frac{1}{4}\;{\text{or}}\; {\raise0.7ex\hbox{${s_{tot ft}^{2} }$} \!\mathord{\left/ {\vphantom {{s_{tot ft}^{2} } {s_{tot lid}^{2} }}}\right.\kern-0pt} \!\lower0.7ex\hbox{${s_{tot lid}^{2} }$}}\; > 4$$

where $s_{tot ft}^{2}$ means *s*_*tot*_^2^ of the FloTrac/Vigileo™, and $s_{tot lid}^{2}$ means *s*_*tot*_^2^ of the LiDCOrapid™. In general, under the null hypothesis that the two variances are equal and their fraction follows an *F*-distribution, the required number of patients was 18 with an alpha of 0.05, and a (1 − β) of 0.8. As this study’s data were aquired using repeated measurements per individual, we concluded that an enrollment of more than 20 subjects would be sufficient.

### Subjects

This research was based on observations performed at a single facility, and examined OPCAB and LDLT patients undergoing planned surgeries between November 2012 and February 2014 (total of 21 cases: 11 OPCAB cases and 10 LDLT cases). The following exclusion criteria were applied during presurgical application: (1) atrial fibrillation (Af) rhythm, (2) emergency surgery, (3) moderate/severe valvular diseases, (4) significant intracardiac shunts, (5) significant arterial occlusions, (6) artificial vessel replacements, (7) massive ascites (>10 % of the body weight), and (8) the presence of any other disease that could influence CO measurements.

### FloTrac/Vigileo^TM^

The FloTrac/Vigileo™ system estimates the CO without correction, using arterial pressure waveforms and the patient’s age, sex, height, and weight. FloTrac/Vigileo™-derived CO is calculated by multiplying the heart rate by the stroke volume. The stroke volume is averaged and displayed every 20 s, and the CO displayed on the monitor represents a 5-min moving average. We used version 3.02 of the software in the present study.

### LiDCOrapid™

The LiDCOrapid™ is similar to the FloTrac/Vigileo™ system in that it facilitates CO measurements without correction using invasive arterial pressure waveforms and the patient’s age, sex, height, and weight. The LiDCOrapid™ uses the PulseCO algorithm, which is characterized by its ability to measure CO per beat. In this research, we used version 1.04-b222 of the software.

### Data collection

All the data were obtained while the patient was in a supine position, and a transducer (TruWave transducer; Edwards Lifesciences) was positioned at the midaxillary line. After an arterial catheter was inserted into the radial artery and attached to the FloTrac™ device, arterial pressure waveforms were sent simultaneously to the Vigileo™ and the LiDCOrapid™. A central venous catheter and a 9-Fr sheath introducer (AK-09903-JJ; Teleflex Inc, Reading, PA) were positioned in the right internal jugular vein, and an 8-Fr PAC (777HF8; Edwards Lifesciences) was inserted through the introducer. The PAC was inserted using the tip pressure as a guide, with confirmation by either chest radiography (LDLT) or transesophageal echography (OPCAB). The CO was measured using a cold saline dilution from the central venous catheter, after confirming that (1) the hemodynamics were stable for 5 min, (2) rapid infusion (>1000 mL/h) was not taking place, and (3) a minimum of 30 min had passed since the previous measurement. The cold water dilution method was implemented using 10 mL of saline cooled to 0 °C with a closed injectate delivery system (CO-Set + ™; Edwards Lifesciences). The temperature of the cooled saline delivered was inputted to the Vigilance™ (Edwards Lifesciences) using a dedicated cable. If the CO measured three times by cold saline dilution showed a disassociation of 15 % or greater, the measured values were discarded. We used the average of three cardiac index (CI) values obtained via the PAC (PAC-CI) as the benchmark. The following sample points were determined in advance:

OPCAB

- (T1) After insertion of the PAC
- (T2) After vertical incision of the sternum
- (T3) During graft harvesting
- (T4) During anastomosis to the LAD
- (T5) During anastomosis to the HL/IM
- (T6) During anastomosis to the LCX
- (T7) During anastomosis to the RCA
- (T8) Before closure of the sternum
- (T9) After closure of the sternum

LDLT

- (U1) After insertion of the PAC
- (U2) Before the start of surgery
- (U3) One hour after the start of surgery
- (U4) Two hours after the start of surgery
- (U5) After complete removal of the liver
- (U6) At the anastomosis of the clamped IVC and the hepatic vein
- (U7) At the anastomosis of the portal vein
- (U8) After recirculation through the portal vein
- (U9) Thirty minutes after reperfusion of the portal vein
- (U10) After anastomosis and recirculation of the hepatic artery
- (U11) After reconstruction of the bile duct
- (U12) At the time of Abdominal closure

At the point at which (1)–(3) were confirmed during surgery, a CO measurement was additionally conducted (Tx, Ux). The FloTrac/Vigileo™ system-derived CI (FT-CI), LiDCOrapid™-derived CI (LiD-CI), mean arterial pressure, central venous pressure, and body temperature measurements were performed before and after the three thermodilutions through the PAC. If the variations of FT-CI, LiD-CI, and mean arterial pressure during thermodilution were 15 % or greater, the measured values were not used in the analysis.

### Statistical analysis

The CI and SVR index (SVRI) were used for the analysis to eliminate the effect of physical size. A Bland–Altman analysis with multiple ovservations per individual was performed, and 95 % confidence intervals (CI) around the LoA were calculated using the method of variance estimates recovery (MOVER) [13]. Furthermore, the trending ability was examined using the polar plot method [14] (cutoff value: 0.5 L/min/m^2^, half-moon method) and a 4 quadrant plot [15] (exclusion zone: 0.5 L/min/m^2^). We used the reported criteria for a good trending ability with an angular bias of no greater than ±5° and a radial limits agreement of no greater than ±30° [14] and a concordance rate of greater than 92 % [16]. To compare the accuracies of the FloTrac/Vigileo™ and the LiDCOrapid™ directly, we assumed that a smaller width of the LoA indicated a superior accuracy, and we compared $s_{tot ft}^{2}$ and $s_{tot lid}^{2}$ using an *F* test. The statistical software package R version 3.2.4 (R: A Language and Environment for Statistical Computing; R Foundation for Statistical Computing, Vienna, Austria) was used.

## Results

**Fig. 1:**
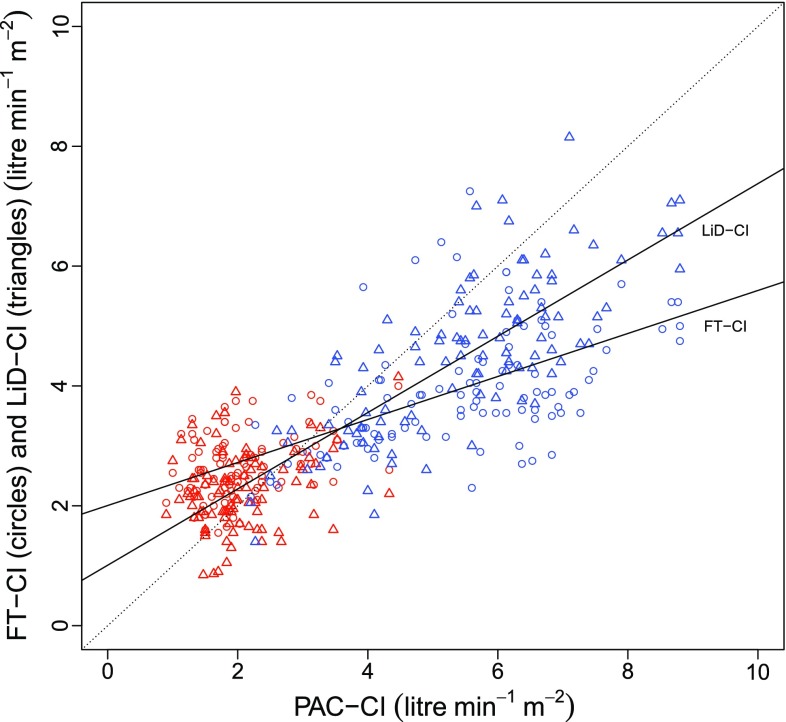
Scatter plot of all data (*red* OPCAB, *blue* LDLT). All analyzed data were plotted with the pulmonary artery derived cardiac index (PAC-CI) on the horizontal axis, the FloTrac/Vigileo™-derived CI (FT-CI) and the LiDCOrapid™-derived CI (LiD-CI) on the vertical axis. Linear regression analysis showed that *y* = 0.36*x* + 2.01 (*R* ^2^ = 0.48) for the FT-CI, and *y* = 0.64*x* + 1.01 (*R* ^2^ = 0.75) for the LiD-CI, respectively

**Fig. 2:**
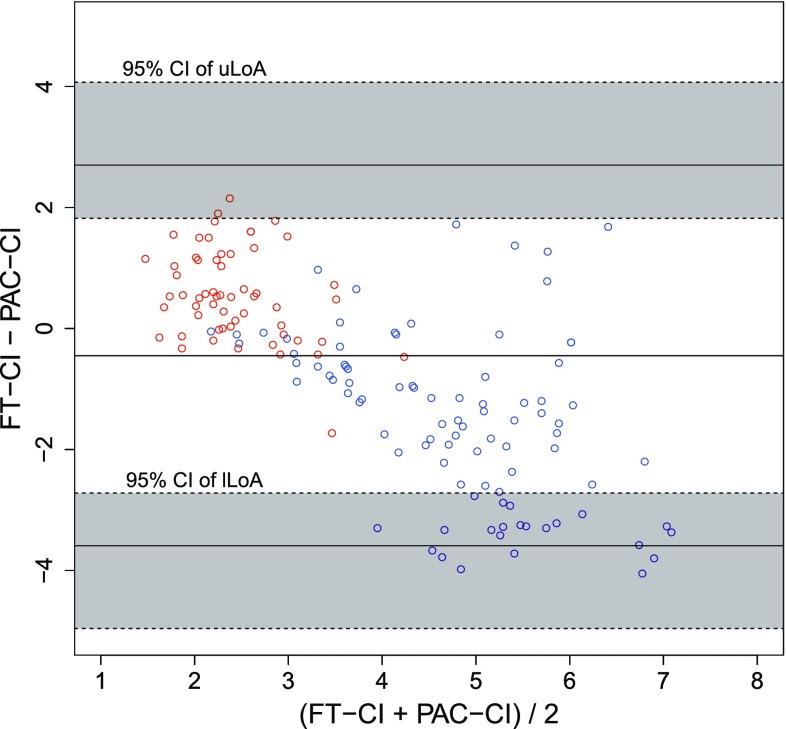
Bland−Altman plot for FT−CI (*red* OPCAB, *blue* LDLT). A Bland–Altman plot of the pulmonary artery catheter measured cardiac index (PAC-CI) and the FloTrac/Vigileo™-derived CI (FT-CI) with 95 % confident interval (CI) of upper limits of agreement (uLoA) and lower limits of agreement (lLoA). In line with the increase in (PAC-CI + FT-CI)/2, FT-CI was estimated to be lower than the actual value (*R* = − 0.71). A Bland–Altman analysis showed the bias (−0.45), percentage error (74.4 %), uLoA (2.70), lLoA (−3.59), 95 % CI of uLoA (from 1.82 to 4.07) and 95 % CI of lLoA (from −4.96 to −2.72)

**Fig. 3:**
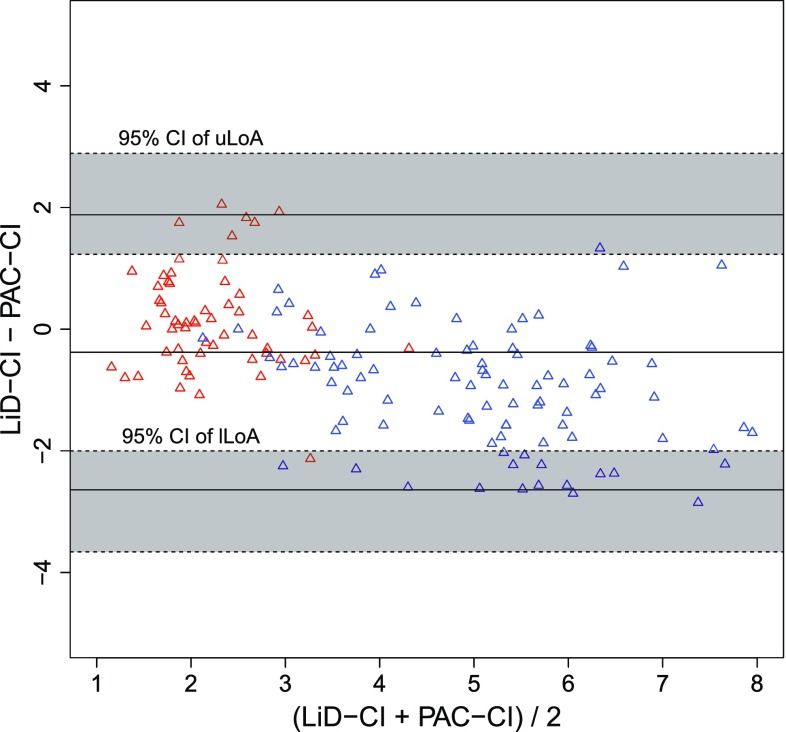
Bland−Altman plot for LiD−CI (*red* OPCAB, *blue* LDLT). A Bland–Altman plot of the pulmonary artery catheter measured cardiac index (PAC-CI) and the LiDCOrapid™-derived CI (LiD-CI). The tendency of underestimation when the increase of (PAC-CI + LiD-CI)/2 was less than that of FloTrac/Vigileo™ (*R* = −0.53). A Bland–Altman analysis showed the bias (−0.38), percentage error (53.5 %), uLoA (1.88), lLoA (−2.64), 95 % CI of uLoA (from 1.23 to 2.89) and 95 % CI of lLoA (from −3.66 to −2.00)

**Fig. 4:**
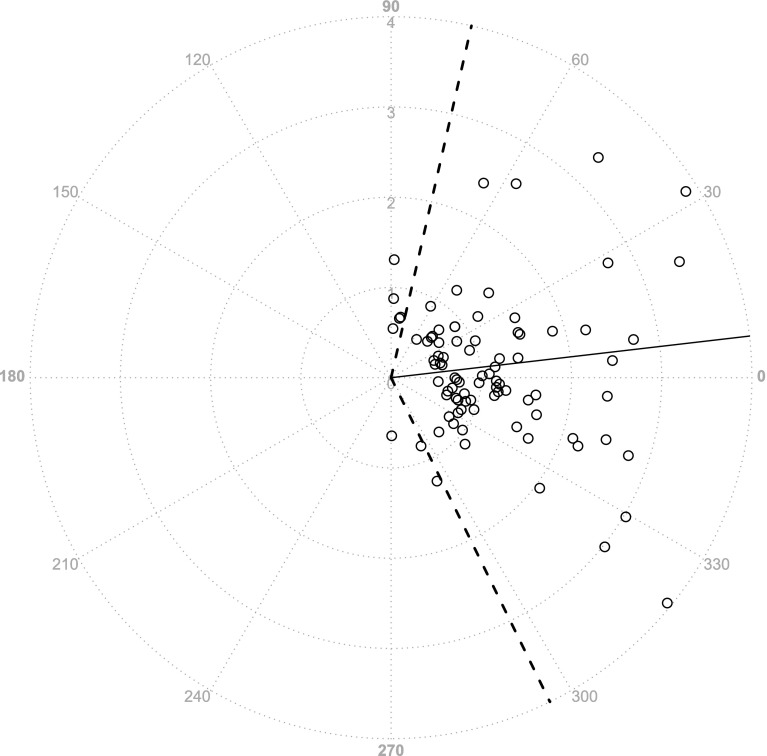
Polar plot method (FT−CI). The trending ability for FloTrac/Vigileo™ was also evaluated by a polar plot method (*n* = 88). The cutoff value was 0.5 L/min/m^2^. The angular bias was 6.6°, and the angular limits of agreement were from −63.9° to 77.1°

**Fig. 5:**
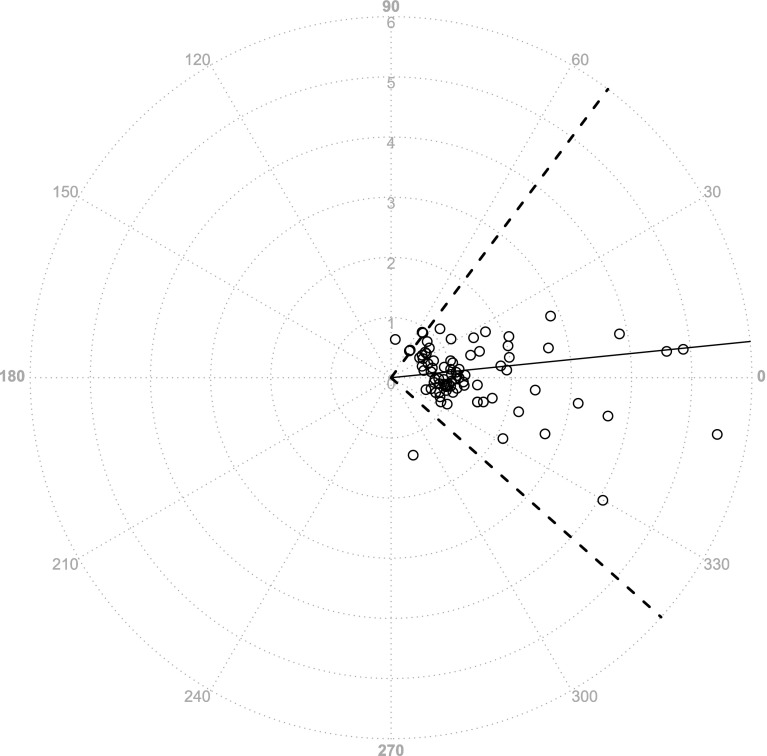
Polar plot method (LiD−CI). A polar plot method for LiDCOrapid™ (*n* = 83). The angular bias was 5.8°, and the angular limits of agreement were from −41.6° to 53.1°

**Fig. 6:**
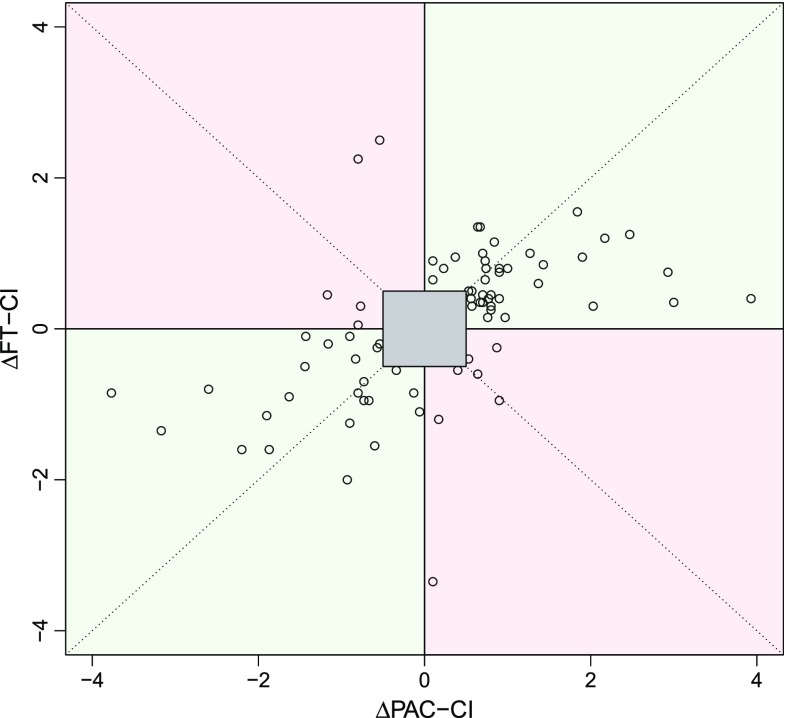
4−Quadrant plot (FT−CI). A 4-quadrant plot for the changes of the pulmonary artery catheter measured cardiac index (∆PAC-CI) and the FloTrac/Vigileo™-derived CI (∆FT-CI) (*n* = 75). When the exclusion zone was set to 0.5 L/min/m^2^, the concordance rate was 84.0 %

**Fig. 7:**
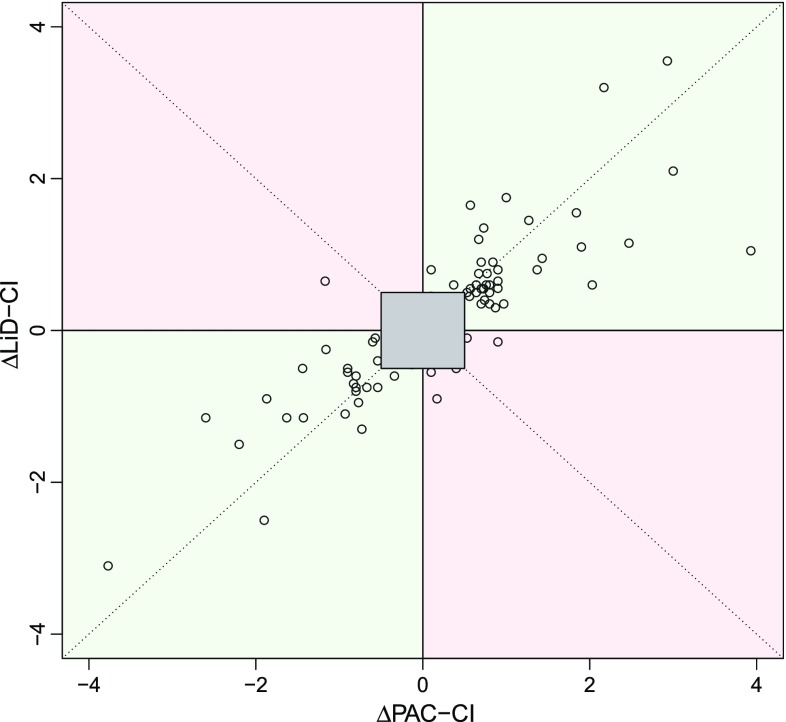
4−Quadrant plot (LiD−CI). A 4-quadrant plot for the changes of the pulmonary artery catheter measured cardiac index (∆PAC-CI) and the LiDCOrapid™-derived CI (∆LiD-CI) (*n* = 79). The concordance rate was 92.4 %

One case of OPCAB required an intra-aortic balloon pump during surgery and was excluded from the analysis. Categorical data were summarized as counts, whereas continuous variables were represented as the median values [interquartile ranges]. The patient background characteristics are shown in Table 1 (overall age, 63.5 [59.3–71.3] years, height, 164.5 [152.2–167.8] cm, weight, 58.0 [52.7–70.5] kg; MELD score for patients undergoing LDLT, 19.0 [13.5–21.0]; and bypass number for patients with OPCAB, 3 [3–5.5]). In total, 91 measurements were obtained from the LDLT patients (9.0 [8.3–10.0] per case, 82 fixed timepoints and 9 other timepoints) and 58 measurements were obtained from the OPCAB cases (5.5 [4.3–7.0] per case, 54 fixed timepoints and 4 other timepoints). When the data were plotted on a graph showing the relationship between PAC-CI and less-invasive-monitors-derived CI, it was clear that a wide range of CI data had been obtained (Fig. 1). The results of a Bland–Altman analysis are shown in Figs. 2 and 3. Neither a logarithmic transformation nor a coordinate transformation was used. For the FloTrac/Vigileo™, the bias and percentage error, which is an indicator of the replaceability [17], were −0.44 L/min/m^2^ and 74.4 %, respectively, for all cases (Fig. 2). For the LiDCOrapid™, the bias and percentage error were −0.38 L/min/m^2^ and 53.5 %, respectively (Fig. 3). The results of the polar plot method are shown in Figs. 4 and 5. The angular bias and radial LoA were 6.6° and −63.9° to 77.1° (FloTrac/Vigileo™), and 5.8° and −41.6° to 53.1° (LiDCOrapid™), respectively; thus, neither of the devices exhibited a good trending ability. The results of a 4-quadrant plot are shown in Figs. 6 and 7. The concordance rate were 84.0 % for the FloTrac/Vigileo™ and 92.4 % for the LiDCOrapid™. When the widths of the LoA were compared using an *F* test, a significant difference was not observed (F-value was $s_{tot ft}^{2} /s_{tot lid}^{2} = 1.39^{2}$, *P* = 0.09).

**Table 1:** Patient demographic data

	LDLT	OPCAB
Age (years)	59.0 [41.2–61.2]	72.0 [65.6–74.0]
Height (cm)	165.0 [151.5–168.5]	164.0 [153.4–167.0]
Weight (kg)	57.2 [52.0–72.8]	58.0 [55.5–70.4]
Sex (male/female)	4/6	7/3
Mean arterial pressure (mm Hg)	62.0 [55.8–66.5]	75.8 [65.2–86.4]
Central venous pressure (mm Hg)	9.0 [7.0–11.0]	9.5 [7.0–12.9]
Mean CI (L/min/m^2^)	5.7 [4.2–6.6]	2.0 [1.7–2.4]
Mean SVRI (dyn s/cm^5^/m^2^)	744 [591–1014]	2685 [2110–3559]

Categorical data were summarized as counts, whereas continuous variables were represented as the median values [interquartile ranges]

*LDLT* living-donor liver transplantation, *OPCAB* off-pump coronary artery bypass graft, *CI* cardiac index, *SVRI* systemic vascular resistance index

## Discussion

This is the first study to includes surgical patients with two physiologically different circulatory conditions, allowing device verification over a broad ranges of CI values (Fig. 1). We hypothesized that performing verification in two conditions that exhibit extremes of both CO and SVR would enable all other circulatory physiological conditions to be interpolated and explained. With FloTrac/Vigileo™, an increased CI was found to produces an accompanying decrease of FT-CI (correlation coefficient in a Bland–Altman plot, *R* = −0.71). This result was consistent with previous reports [4–8], suggesting that the expectations of this study were met. A similar relationship was noted using LiDCOrapid™, although the relationship was weaker (correlation coefficient in a Bland–Altman plot, *R* = −0.53) than that identified using the FloTrac/Vigileo™. Under these conditions, neither the FloTrac/Vigileo™ system nor the LiDCOrapid™ achieved replaceability according to Critchley’s criteria [17]. An *F* test revealed that there was no significant difference in the widths of LoA between the two devices when a sample size capable of detecting a two-fold difference was used. As previously reported [4], the FloTrac/Vigileo™ system showed a broad radial LoA (from −63.9° to 77.1°) and its trending ability was found to be low. These results were supported by the concordance rate for the FloTrac/Vigileo™ (84.0 %). On the other hand, LiDCOrapid™ had a lower radial LoA (from −41.6° to 53.1°) and a larger concordance rate (92.0 %) than the FloTrac/Vigileo™ system. For both the FT-CI and LiD-CI, a tendency to underestimate CI in line with increased CI was observed. These results suggest that this may be a problem unique to CO measurement using arterial pressure waveform analyses.

The three types of CO measurement methods require different durations for completing CO measurement. Using the thermodilution method via a PAC, the CO was obtained within approximately 3 min, which was the time required from the start of the measurement until completion, and represented the average CO for that 3-min period. The FloTrac/Vigileo™ system determines two parameters calculated at 20 s and 1 min, respectively, before displaying the measured value. The LiDCOrapid™ measures the CO per heartbeat. To compare these three types of CO measurements, we decided to exclude any data with a variation of 15 % or more in the measurement parameters between the point when thermodilution was started after the hemodynamics had been stable for 5-min or more and the point when thermodilution was completed. Furthermore, the CIs of the FloTrac/Vigileo™ system and LiDCOrapid™ were averaged before and after thermodilution.

### Limitations

The present study had several limitations. First, the study was performed at a single institution, and all the subjects were Japanese. As a result, it is impossible to rule out the effect of the facility on the applicability of OPCAB and LDLT in this study, and our results might only be applicable to Asian people. Next, the patients involved had a particular circulatory physiology (either OPCAB or LDLT). Although we believe that our results can be interpolated to patients in general (i.e., those with a normal CI, and a normal SVRI), caution is needed because we did not directly compare PAC attached high risk patients and low or intermediate risk patients [18]. Furthermore, it was not possible to clarify whether CO or blood vessel resistance had a greater impact on either the FloTrac/Vigileo™ system or the LiDCOrapid™, because the subjects were surgical patients, and from the perspective of maintaining a mean arterial pressure, we could not manipulate the CO and SVR independently.

## Conclusion

We could compare CO measurement devices across broad ranges of CO and SVR using LDLT and OPCAB surgical patients. An *F* test revealed that there was no significant difference in the widths of the LoA for the both devices when a sample size capable of detecting a two-fold difference in the width of the LoA was used. We found that both devices tended to underestimate the calculated CIs when the CIs were relatively high. These proportional bias produced large percentage errors in the present study.
